# The Story of the Hospitals

**Published:** 1886-12-25

**Authors:** 


### THE STORY OF THE HOSPITALS.

By One of the Public.

(*Continued from page* 158.)

### The Royal Free Hospital.

The First Free Hospital.

The Royal Free Hospital, Gray's Inn Road, was founded by Dr. William Marsden (whom we shall notice among our "Hospital Worthies") in 1828, on a principle never before attempted, namely, that of making a hospital accessible, as far as its means would allow, to every diseased and destitute person who applied for succour at its doors, without any recommendation other than his necessities. Previously to the establishment of this institution no person could obtain admission into the wards of a hospital, or be the recipient of its benefits as an out-patient, without first having procured a governor's letter; that is to say, a letter from some privileged person or subscriber recommending the patient, and, in some cases, an undertaking from a householder or ratepayer to receive him on leaving the institution or to bury him if he died! The obstacles opposed by the necessity of these letters to prompt reception and speedy relief were frequently found (in cases most urgently demanding both) to act as a complete prohibition.

A Touching Incident.

In the winter of 1827 a wretched girl, only some 18 years of age, was observed by Mr. Marsden, as he was journeying homewards one midnight, lying prostrate on the steps of St. Andrew's churchyard in Holborn Hill, actually perishing through disease and famine. He conveyed her in a hackney coach to three or four of the large hospitals, with a hope of obtaining admission, but not being provided with the necessary letters, they all refused her, and so it seemed she must die upon the hard streets. Marsden felt shame and indignation at such a state of things as this, and he determined it should exist no longer. Disease and destitution, he contended, should be the only passports to the hospital. The poor dying girl, who was friendless in London, was eventually taken by Mr. Marsden to a lodging, where, two days afterwards, she died, unrecognised by a human being.

The Royal Free Founded.

This distressing event, although by no means one of unfrequent occurrence at that period, determined William Marsden to found a hospital on the principle of free and instant admission, in order to provide, if possible, for all cases of a similar nature. This idea he communicated to some of his neighbours and friends, among whom were Mr. Alderman Harmer (at whose residence the early meetings of the institution were held), Alderman Winchester, Mr. John Watson, of Holborn Hill, and others, who with small beginnings and still smaller means, opened a hospital or dispensary on this plan, in Greville Street, Hatton Garden, under the name of "The London General Institution for the Gratuitous Cure of Malignant Diseases." Many of those who supported the founder in his design regarded the success of the undertaking as very doubtful, and some even asserted that the principle could never be carried out. In face, however, of many difficulties and the opposition of many prejudices, it continued to advance in public favour, and to extend the sphere of its usefulness. Through the influence of Sir Robert Peel, whose aid Marsden sought, the patronage of King George the Fourth was conceded to it, and in the following year the Duke of Gloucester became its president. The Corporation of the City of London presented several munificent donations towards its support, and the countenance and aid of other public bodies, and of many noblemen and distinguished personages, were obtained.

The Cholera of 1832.

While the Royal Free was yet in its infancy a terrible scourge—malignant cholera—appeared in London. In order to carry out to the fullest extent the great principle of the charity, the Governors, at Marsden's suggestion, at once threw open the doors of the hospital to all persons afflicted with that dreadful malady, and this, too, notwithstanding the fact that the other hospitals had closed theirs against them. Upwards of 700 cholera patients were admitted, and the funds of the charity became exhausted; but as the demands on the usefulness of the institution continued to increase, so did its means, till, finally, the premises in Greville Street were no longer sufficient to meet the demands made upon them.

The Hospital migrates.

So the Governors cast about for another building. They applied to Government for pecuniary assistance and a grant of land for a site for a new hospital, but their application did not succeed. Some buildings in the Gray's Inn Road, formerly occupied by the Light Horse Volunteers, having become vacant, the Governors at once resolved to take them and convert them into a hospital, both the situation and the building itself being admirably adapted for the purpose required. In this step they received the hearty concurrence and support of the late Marquis of Westminster, Lord Robert Grosvenor, the Earl of Arundel and Surrey, Lord Calthorpe, Viscount Blake, and many other noblemen and gentlemen. A meeting was held in Willis's Rooms in the spring of 1843, for the purpose of raising means to meet the necessary outlay. A fund was started, and among the first subscribers we find the names of the Queen (now patron of the hospital), Prince Albert, and the Queen Dowager Adelaide.

The New Hospital.

A contemporary journal thus describes the hospital at the time it was opened: "It is a very handsome piece of masonry, and consists of a central arch surmounted by the British lion, and two wings extending north and south. The north wing contains two spacious and airy wards, the board-room, and counting-house. The south wing comprises one ward, the physicians' and surgeons' consulting-rooms, with waiting-rooms adjoining for the patients, the men and women being kept separate, and the dispensary to which they proceed on getting their prescriptions from the medical gentlemen and at once obtain their medicine, everything being done with a precision almost military. The internal management is excellent, the wards are patterns of neatness and cleanliness, of comfort and convenience; and that person must possess little of humanity who could pass through them and behold the hungry fed, the naked clothed, and the sick visited, without a swell of heart to which words would but imperfectly give utterance."

A Testimonial to the Founder.

It is pleasing to be able to record the fact that the noble philanthropy which stirred the breast of Dr. William Marsden did not pass without just recognition. His friends—and they grew to be legion—determined that their appreciation of his grand work should find a fitting expression. The testimonial, which was presented to the founder of the Royal Free Hospital by the Duke of Cambridge, comprised an elegant centre-piece, two wine coolers, and four dishes. "The centre-piece consists of three figures resembling the Graces, beautifully executed in frosted silver, standing upon a triangular base, and bearing upon their raised arms a filigree basket for fruit or flowers. On one side of the triangle is a bas-relief representation of the Scripture parable of the Good Samaritan. The traveller who fell among thieves has been left to perish, and the priest and Levite have passed by on the other side; but the Good Samaritan is seen binding up his wounds and pouring in oil and wine. On another side are the arms of the founder, and on the third, an appropriate inscription." The journal from which we have quoted adds: "The plate of which this splendid testimonial consists cost no less a sum than £800, and the subscription list numbered upwards of 300 persons, the amount of subscriptions varying from 10s. to £5, the latter being the maximum sum allowed to be contributed. There is scarcely another instance on record of a private individual having received from his friends and the public so magnificent and costly a testimonial of their approbation, and the alacrity with which the money was subscribed showed how strongly they felt the deserts of the recipient. Indeed, the founder of this invaluable institution displayed no ordinary degree of moral courage in carrying out his plan, for he made it triumph not by means of station or influence, but by his genius, talent, perseverance and pecuniary sacrifice." He convinced the public of the soundness of the principle, and that once accomplished, the charity soon found friends in every quarter, although in the struggle of its earlier years some of the members of the managing committee were so fearful for its success that they actually withdrew from the responsibility, and the establishment must have fallen to the ground had not the founder taken the whole risk on his own shoulders.

Onward Progress.

At this period the hospital contained five wards, and could accommodate 150 in-patients. From that time to the present, with varying fortune, the hospital has gradually increased in public favour, and received more and more support. The freehold, we may add, was bought through the exertions of the late George Moore and others, and the generosity of the late Lord Calthorpe, the freeholder, who sold the land and buildings at a remarkably low figure.

Donations by stealth.

At the hospital gate the visitor will notice a subscription box, but, unlike most subscription boxes, it used in olden days to become the receptacle of donations of unusual magnitude. To do good by stealth is not, as a rule, the injunction we act up to in 1886, but forty years ago there were men who occasionally made the box worth opening. On 27th December 1843, a bank-note was found, value £100, and marked "A Passer-by." On 14th June 1844, another note, of similar value, was deposited by another "Passer-by." On the 2nd November following a further hundred pounds was found, with a piece of paper bearing the words, "Winter is coming on—*Bis dat qui cito dat.*" On 9th October 1850 a fifty-pound note was found, and on 21st June 1851 one for twenty pounds. Nowadays the box reveals no such "finds," although the Royal Free Hospital was never perhaps in greater need of support, and probably never deserved it more than at present.

